# Antibacterial efficacy of non-thermal atmospheric plasma against *Streptococcus mutans* biofilm grown on the surfaces of restorative resin composites

**DOI:** 10.1038/s41598-021-03192-0

**Published:** 2021-12-10

**Authors:** Gabriel Nima, Erika Harth-Chu, Rochelle Denise Hiers, Vanessa Gallego Arias Pecorari, David W. Dyer, Sharukh Soli Khajotia, Marcelo Giannini, Fernando Luis Esteban Florez

**Affiliations:** 1 Department of Restorative Dentistry, Dental Materials Division, Piracicaba Dental School, State University of Campinas, Piracicaba, SP Brazil; 2 Department of Oral Diagnosis, Piracicaba Dental School, State University of Campinas, Piracicaba, SP Brazil; 3 Department of Restorative Sciences, Division of Dental Biomaterials, College of Dentistry, The University of Oklahoma Health Sciences Center, Oklahoma City, OK USA; 4 São Paulo Dental School, Paulista University, São Paulo, SP Brazil; 5 Department of Microbiology and Immunology, College of Medicine, The University of Oklahoma Health Sciences Center, Oklahoma City, OK USA; 6 Department of Restorative Dentistry, Operative Dentistry Division, Piracicaba Dental School, State University of Campinas, Piracicaba, SP Brazil

**Keywords:** Dental caries, Microbiology, Materials science

## Abstract

The aim of this study was to evaluate the antimicrobial efficacy of non-thermal atmospheric plasma (NTAP) against *Streptococcus mutans* biofilms. Resin discs were fabricated, wet-polished, UV sterilized, and immersed in water for monomer extraction (37 °C, 24 h). Biofilms of bioluminescent *S. mutans* strain JM10 was grown on resin discs in anaerobic conditions for (37 °C, 24 h). Discs were divided into seven groups: control (CON), 2% chlorhexidine (CHX), only argon gas 150 s (ARG) and four NTAP treatments (30 s, 90 s, 120 s, 150 s). NTAP was applied using a plasma jet device. After treatment, biofilms were analyzed through the counting of viable colonies (CFU), bioluminescence assay (BL), scanning electron microscopy (SEM), and polymerase chain reaction (PCR). All NTAP-treated biofilm yielded a significant CFU reduction when compared to ARG and CON. BL values showed that NTAP treatment for 90 s, 120 s or 150 s resulted in statistically significantly lower metabolic activity when compared to the other groups. CHX displayed the lowest means of CFU and BL. SEM showed significant morphological changes in NTAP-treated biofilm. PCR indicated damage to the DNA structure after NTAP treatment. NTAP treatment was effective in lowering the viability and metabolism of *S. mutans* in a time-dependent manner, suggesting its use as an intraoral surface-decontamination strategy.

## Introduction

Biofilms are complex communities of microorganism attached to a substratum or to an interface, composed by hundreds of multiples species that interacts between them^1^. In biofilms, cells are embedded in a self-produced matrix of extracellular polymeric substances, that not only act as a physical barrier to external aggressors^1,2^, but also permit efficient cooperation among community cells^3^ and the storage of nutrients^4^. Biofilms are involved in the etiology of the most common oral diseases including dental caries, periodontal disease, and periimplantitis^5^.

*Streptococcus mutans* has been implicated as the major causative agent of primary and recurrent caries^6,7^. *S. mutans* has been used as a cariogenic model organism for many years due to its ability to metabolize sugars into acids, and because this microorganism is capable of forming biofilm structure by the production of water-insoluble glucans^8^.

The clinical management of dental caries involves the removal of disorganized and bacteria-contaminated hard tissues using low-speed carbide burs under abundant irrigation with air/water spray, followed by the placement of restorative resin composite (either direct or indirect)^9^. However, despite the success and widespread utilization of such technique, an additional healthy dental hard tissue is typically lost during this process. Such extra tissue removal is made necessary to biomechanically prepare the tooth structure to receive and retain the non-adhesive restorations, to finish the preparation margins, and to maintain the long-term biological and mechanical integrity of the tooth structure^10^. Various antibacterial agents, such as chlorhexidine, sodium hypochlorite, and benzalkonium chloride have been used after the completion of cavity preparation procedures to control the viability of the bacterial load present^11,12^. However, even though the antibacterial agents cited were effective against oral bacteria, some adverse effects have been reported on dentin bond strength of dental adhesives^13,14^, marginal staining, microleakage and development of bacterial resistance^15,16^.

In this critical scenario, the development of alternative approaches and techniques that are capable of effectively decontaminating non-shedding intraoral surfaces (either biotic or abiotic) effectively, fast and without promoting the development of antibiotic resistance are of importance in dentistry. One possible alternative to overcome the limitations cited is the utilization of non-thermal atmospheric plasma (NTAP). Typically, NTAP is generated by the application of energy to an inert gas such as helium, argon or neon. The physical manifestation of such process, is the promotion of electrons from the fundamental state into a higher energy state^17^, with the subsequent formation of ions, electrons, protons, UV-radiation and reactive species of nitrogen (RNS) and oxygen (ROS)^10,18,19^. Therefore, this room-temperature and minimally invasive approach has been used in several industrial segments and health care-related fields including automobile, engineering, transport, electronics, food packing, cancer treatment, blood coagulation, cosmetic treatments, ophthalmology, urology and cardiology^20–22^. In dentistry, plasma has been used for surface decontamination^10,21,23^, improving the implant osseointegration^24,25^, enhancing the adhesion of polymeric materials to dentin^26–28^, increasing the surface energy, wettability and adhesion to dental materials^29–31^, and enhancing the tooth whitening effects^32–34^.

According to previous reports the antibacterial properties of the NTAP has been primarily correlated to NTAP’s ability to generate RNS and ROS^10,21,23,35^. Delben et al. have investigated the effect of NTAP against dual-species oral biofilms (*Candida albicans* and *Staphylococcus aureus*) and demonstrated that NTAP is effective in destroying and removing pathogenic biofilms^18^. Matthes et al.^36^ evaluated the antimicrobial capacity of NTAP against Pseudomonas aeruginosa and Staphylococcus epidermidis biofilms founding that it was at least as effective as chlorhexidine and the antibacterial effect depends on the application time and the bacteria. Another study using confocal microscopy demonstrated that plasma significantly reduce the viability of *Streptococcus mutans, Streptococcus sanguinis,* and *Streptococcus gordonii*^37^. Many studies available^38–41^ have investigated the use of a vacuum chamber or atmospheric glow discharge-generated low-pressure plasma. One of these studies^38^ showed bacterial growth up to 3 logs using a direct current corona discharge plasma with a 10-min exposure time. Even though these are highly efficient systems, they cannot be used intra-orally due to technological restrictions^42,43^.

Thus, the purpose of this study was to evaluate the antimicrobial efficacy of NTAP using a plasma jet configuration against non-disrupted biofilms of *S. mutans* grown on restorative composite. The null hypotheses tested were: (1) NTAP would not present an antibacterial effect against biofilms of *S. mutans* grown on resin composite*,* (2) NTAP would not display time-dependent antibacterial effects, (3) NTAP would not significantly alters the structure and morphology of *S. mutans* biofilms and (4) NTAP would not be capable of producing damage to the DNA of *S. mutans* biofilms.

## Materials and methods

### Specimen fabrication

Disc shaped sample (diameter = 7.00 mm, thickness = 0.8 mm) were fabricated using the resin composite Point 4™ (shade A2, Kerr Corp., Brea, CA, USA) and a custom-made metallic mold. Samples were light cured (40 s/specimen) using an LED light-curing unit (Valo, Ultradent Products Inc.; South Jordan, UT, USA) before being sequentially finished and polished (180–1200 grit SiC, final polish = 0.5 µm, diamond aqueous suspension) under copious water irrigation in a semi-automated grinder-polisher (MultiPrep TM, Allied High-Tech Products Inc., Compton, CA, USA) following a previous protocol from our laboratory^44^. Polished specimens were then UV-sterilized in a crosslinker (254 nm, 800,000 mJ/cm^2^, CL-1000 UVP, Analytik Jena US LLC, Upland, CA, USA) and stored in ultra-pure water for monomer extraction (37 °C, 24 h).

### In vitro growth of biofilms

Overnight cultures of *Streptococcus mutans* JM10 were grown (18 h, 37 °C, static and anaerobically) in THY culture medium supplemented with 0.3% yeast extract (Y) and spectinomycin (100 mg/mL). Overnight cultures in THY with OD_600_ ≥ 0.900 (corresponding to 6.43 e^+12^ CFU/mL)^45^ were used as inoculum for biofilm grown in our experiments. Biofilms were formed by mixing a 1:500 dilution of an overnight culture in 0.65 × THY medium supplemented with 0.1% (w/v) sucrose. Aliquots (2.5 mL) were individually dispensed into separate wells of sterile 12-well plates (Falcon, Corning, Corning, NY, USA) containing the sterile and monomer-extracted specimens. Biofilms were then grown against the surfaces of disc-shaped composite samples for 24 h (static cultures, anaerobic conditions, 37 °C). After 24 h of incubation, specimens were carefully transferred to sterile 12-well plates and washed two times with 2 mL of sterile saline solution.

### Plasma generation

Non-thermal atmospheric plasma was generated using the hand-held unit (electromagnetic generator; power output: 45 W, frequency: ~ 30 kHz, Tension: 110 V) of a commercially available plasma-generating device (Surface Plasma Tool Model SAP; Surface–Engineering and Plasma Solution; Campinas, SP, Brazil) from the ionization of argon gas (flow rate: 8 L/minute) at normal conditions of temperature and pressure. Detailed information regarding the plasma-generating device can be found in a previous publication^46^.

### Antibacterial treatments

Disc-shaped composite samples were subjected to the conditions and antibacterial treatments. One group of specimens were used as growth control (CON) and no treatment was applied on them, specimens of chlorhexidine (CHX) group were exposed to 2% Chlorhexidine for 1 min^47^. Chlorhexidine is an effective antibacterial agent against biofilms of *S. mutans*^48,49^.

Composite samples of the other groups were subjected to experimental antibacterial treatments with only argon gas for 150 s (ARG – plasma generator off) or plasma [treatment times: 30 s (P30), 90 s (P90), 120 s (P120) and 150 s (P150)] generated by the hand-held unit (fixed height from specimens: 10.00 mm) of the plasma-generating device. For ARG group the argon was not ionized (plasma generator off). The samples were kept moistened during plasma application. To guarantee that samples were kept moist, 20 µl of saline solution was added to each sample every 60 s. A single and previously calibrated operator moved in a sweeping motion the petri dish (over a fixed surface) containing a single specimen to completely treat the surface of each specimen.

### Viable colony counts (VCC)

A set of disc-shaped composite samples (n = 4/group) was subjected to biofilm growth and antibacterial procedures as described in “Specimen fabrication” to “Viable colony counts (VCC)” section. After the completion of investigated antibacterial treatments and incubation in recharge medium (1 h, 37 °C), biofilms were sonicated (Sonicator 3000, Misonix Incorporated, Farmingdale, NY, USA) to allow the efficient and reproducible detachment of bacterial biomass from the surface’s specimens. Aliquots (10 µL) of bacterial suspensions were then serially diluted (10^–8^). Finally, aliquots (10 µL) of each dilution produced were plated in triplicate on THY plates supplemented with streptomycin (800 µg/mL) and incubated for 48 h (37 °C, anaerobic conditions). After the incubation period, VCC (in CFU/mL) were visually determined and calculated. The experiment was repeated four times.

### Bioluminescence assay

In order to determine the metabolic status of *S. mutans* biofilms, the present study utilized a real-time and high throughput bioluminescence assay^45^. The metabolic status of *S. mutans* biofilms was determined at two distinct and subsequent time periods (baseline [BR] and after antibacterial treatments [AR]) to determine the antibacterial efficacy of treatments proposed (in terms of relative luminescence units [RLU]).

A set of disc-shaped composite samples (n = 4/group) was subjected to biofilm growth and antibacterial procedures as described in “Specimen fabrication” to “Viable colony counts (VCC)” section. After the biofilm growth period, composite samples with 24-h biofilms were individually and carefully transferred into separate wells of sterile 24-well plates containing 1 mL of 1 × THY + 1% (w/v) glucose (recharge medium) and were incubated for 1 h (37 °C) to replenish their internal energy levels in preparation for the first round of high throughput bioluminescence testing. The BR bioluminescence measurement was performed immediately after individually dispensing D-Luciferin aqueous solution (500 µL, 100 mM) suspended in 0.1 M citrate buffer (pH 6.0) into each well using a computer-controlled system in a Synergy HT Multi-mode microplate reader (BioTek Instruments, Inc., Winooski, VT, USA). The assessment of the temporal evolution of luciferase metabolic activity in non-disrupted biofilms of *S. mutans* was performed in 2-min increments (6 min total; BR0–BR6) after the addition of D-Luciferin substrate at 530 nm. Afterwards, the media of each well was carefully aspired and replace with fresh new medium (1 mL of 0.65 × THY + 0.1% (w/v) sucrose). Disc-shaped composite samples were then randomly assigned into seven experimental groups. Before being subjected to experimental treatments, the second round of bioluminescence assay (AR0–AR6) was performed under the same conditions used for the first reading.

### Morphological examination

Scanning electron microscopy (JSM-5600LV; JEOL, Tokyo, Japan) was used to reveal the morphological and structural features observed on non-disrupted biofilms of *S. mutans*, and after antibacterial treatments. An additional set of disc-shaped composite samples (n = 2/group) was subjected to the procedures described in “Specimen fabrication” to “Viable colony counts (VCC)” section. Samples were then fixed (2 h, 4 °C) with an aqueous solution of 2.5% glutaraldehyde (Sigma-Aldrich Corp; St Louis, MO, USA) before being subjected to sequential ethanol dehydration (From 50 to 100%; 10 min/step). After dehydration, samples were mounted onto metal stubs and were sputter-coated with gold (Desk II, Denton Vacuum Inc.; Moorestown, NJ, USA) then observed at 500× and 3000× magnification.

### DNA extraction and polymerase chain reaction

Immediately after the viable colony counts assay, aliquots (990 μL) of sonicated bacteria suspended in recharge medium were transferred to microcentrifuge tubes in preparation for DNA extraction and polymerase chain reaction (PCR). Microcentrifuge tubes were centrifuged (13,000 relative centrifugal force 4 °C) for 2 min. Following, the supernatant was carefully aspirated to obtain a pellet of *S. mutans*, the genomic DNA was extracted using the Wizard Genomic DNA Purification Kit (Promega, Madison, WI, USA). The amount of DNA obtained was quantified photometrically (absorbance) at 260 nm (NanoDrop ND-1000 spectrophotometer, Thermofisher, Waltham, MA, USA). The amplification of the 16S rRNA was carried out using the primer listed in Table 1. For any given sample, PCR reactions contained Mastermix (12.5 µL; GoTaq Colorless Mastermix, Promega, Madison, WI USA), primer (0.75 µL, 10 nmol), template DNA (1 µL, corresponding to 280 ng) and sterile ultrapure water (10 µL). The four-step amplification program (step 1 – initial denaturation = 1 cycle of 3 min at 94 °C, step 2 – denaturation = 30 cycles of 30 s/each at 94 °C, step 3 – annealing = 1 cycle of 30 s at 56 °C and step 4 – extension = 1 cycle of 10 min at 72 °C) was carried out in a Mastercycler® Gradient (Eppendorf, Endfield, CT, USA). To confirm successful amplification, electrophoresis procedures were conducted in agarose gel (0.9%) with added ethidium bromide (Sigma-Aldrich Corp; St Louis, MO, USA). Aliquots (15 µL) of PCR product samples were then loaded into separate wells using Loading Dye (Purple [6x], New England Biolabs, Ipswich, MA, USA) and subjected to electrophoresis for 45 min (110 V). The experiment was repeated four times.

**Table 1:** Oligonucleotides used in this study.

Primer name	Sequence 5′-3′	Product size (bp)	Source
16SmutansF	GGGTGAGTAACGCGTAGGTA	1401	This study
16SmutansR	TGTTACGACTTCACCCCAAT		

### Statistical analysis

The VCC data were tested for normality and homogeneity using Shapiro-Wilks and Brown-Forsythe test respectively at a significance level of 95% (α = 0.05). Since data was not normally distributed, experimental data was then corrected using the Box-Cox method (*p* = 0.6025). Corrected data was then analyzed using one-way ANOVA and Dunnett’s post hoc test (α = 0.05). Additionally, for NTAP groups, ANOVA and Tukey’s post hoc tests, and polynomial regression were performed (α = 0.05).

For bioluminescence data, the calculation of the relative metabolic differences within each group were performed according to equation 1^50^ below:

$${Met}_{diff}=\frac{({Met}_{f}-{Met}_{i})}{{Met}_{i}}$$

where *Met*_*diff*_ stands for relative metabolic difference, *Met*_*i*_ stands for metabolic activity at baseline (BR0, BR2, BR4 and BR6) and *Met*_*f*_ stands for metabolic activity after treatments (AR0, AR2, AR4 and AR6). Then, data were tested for normality and homogeneity of variances using Shapiro-Wilks test (α = 0.05). Since data was not normally distributed (*p* < 0.05), the logistic model PROC GENMOD and the Wald test were used to determine the existence of significant differences among mean values of relative metabolic differences.

All the statistical analyses were performed using SAS software for windows (Version 9.3; SAS Institute, NC, USA).

## Results

The mean and standard deviation values of VCC are summarized on Table 2. One-way ANOVA showed significant differences between the groups (*p* < 0.0001), Dunnett’s post hoc demonstrates statistical differences of the controls (CON and CHX) with the ARG and all the plasma groups. P120 and P150 were the most effective treatments and reduced the biofilm viability with a killing rate of 35.25% and 38.3% respectively, with statistical differences regarding other groups according to the Tukey’s test. Plasma effect was time-dependent; the most efficient deactivation time was P150 but a few CFU reduction was observed between P120 and P150 groups (Table 2).

**Table 2:** Effect of plasma application on *S.mutans* biofilm (Log CFU/ml).

Groups	CFU log/ml	Log reduction	Percentage reduction	ANOVA test
Control	9.19 ± 0.45	Reference	Reference	A
Plasma 30”	7.42 ± 0.48	1.77	19.2%	B
Plasma 90”	6.80 ± 0.25	2.39	26%	C
Plasma 120”	5.95 ± 0.12	3.24	35.25%	D
Plasma 150”	5.67 ± 0.4	3.52	38.3%	D
Argon gas	8.20 ± 0.35	0.99	10.8%	*
Chlorhexidine 2%	0	9.19	100%	*

Groups having similar letters are not significantly differences (*p* > 0.05), *Indicates significantly differences with all the groups.

**Figure 1:**
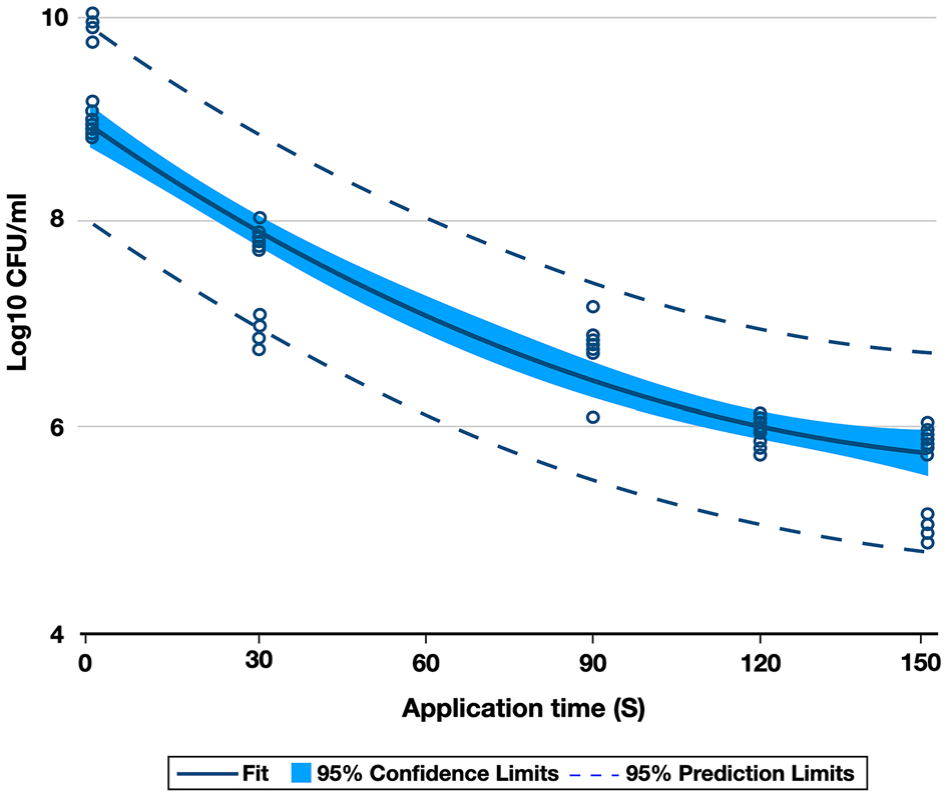
Polynomial regression of the CFU of the *S. mutans* biofilm treated with NTAP in different times (s).

Polynomial regression (Fig. 1) showed significance (*p* < 0.05) for the quadratic polynomial, where NTAP displayed a time-dependent antibacterial effect against biofilms of *S. mutans*. Moreover, experimental data indicate that application time (30–150 s) has an exponential relation to the reduction of bacterial viability and reaches its maximum antibacterial effect at 120 s. The results clearly demonstrated a time-depend antibacterial effect of the plasma.

**Figure 2:**
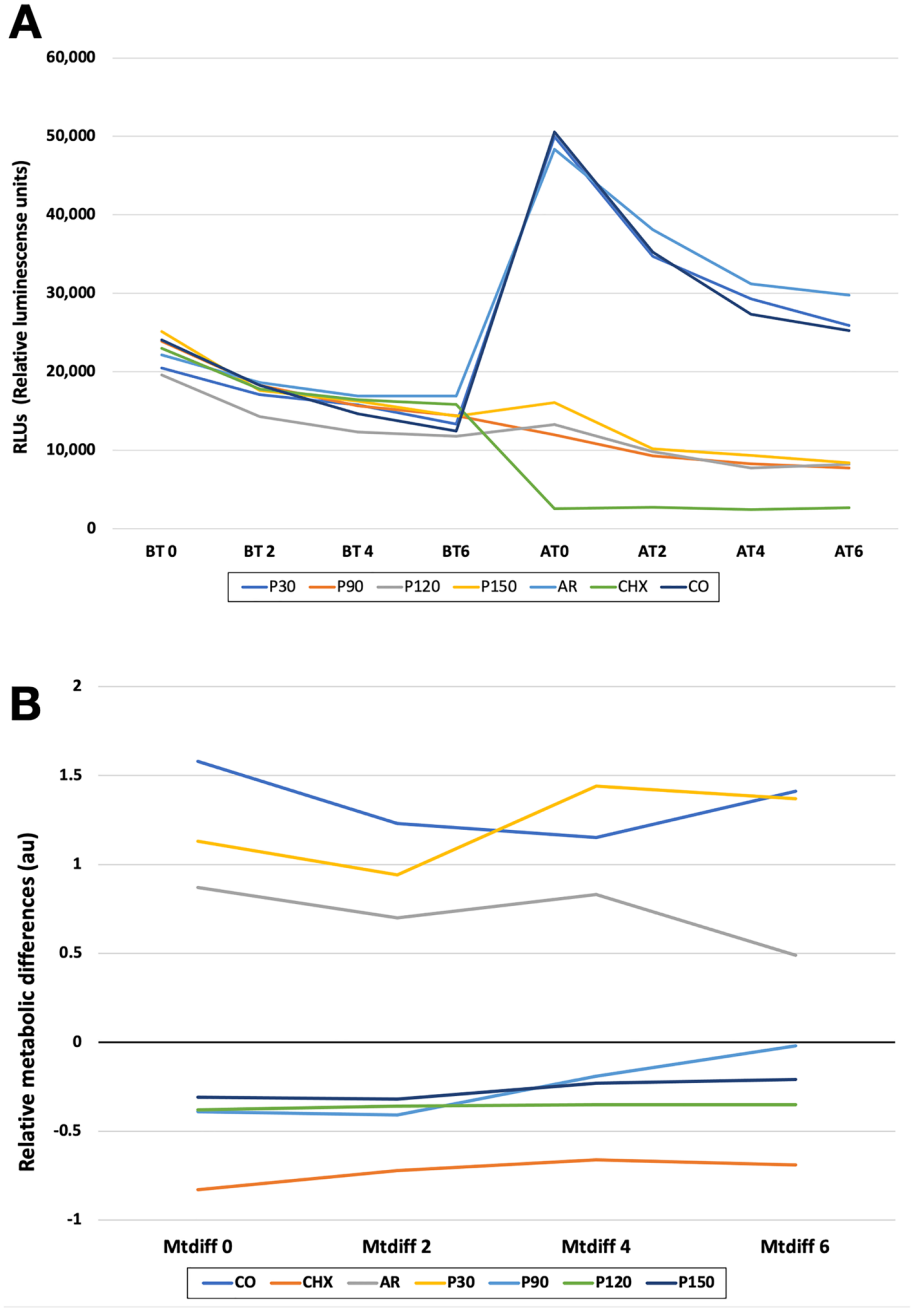
Antibacterial efficacy of experimental treatments against *Streptococcus mutans* biofilms based: (**A**) Metabolic activity in terms of Relative Luminiscense Units (RLU) of the *S. mutans* biofilms before (BT0–BT6) and after (AT0–AT6) experimental treatments; and (**B**) Metabolic activity of the experimental treatments against *S. mutans* biofilms in terms of metabolic activity differences calculated for each point time. The groups P90, P120 and P150 had antibacterial effect. However, CHX was the most antibacterial treatment. P30, AR and CO groups showed higher metabolic activity.

The metabolic activity of luciferase in *S. mutans* biofilms in terms of relative luminescence units are showed in Fig. 2A. It was measured in the evolution of time in 2-min increments. Thus, four-measure points before (BT0 – BT6) and after (AT0 – AT6) experimental treatments were evaluated. The results show that all the experimental groups had similar metabolic activity before the treatment application. After treatment application, some differences in the metabolic activity of the groups were observed. The most efficient antibacterial treatment was CHX followed by P90, P120 and P150 groups. The ARG and P30 groups showed higher metabolic activity close to CON group.

The median values of the temporal evolution of relative D-Luciferin metabolic activity in the experimental groups determined by the bioluminescence assay in terms of relative metabolic differences are shown in Table 3. The lower D-Luciferin metabolic activity among experimental groups was observed in groups P90, P120 and P150. The statistical analysis showed that the factor “treatment” was a significant (*p* = 0.048) predictor of response (e.g., metabolic activity) and influenced RLU values. However, the factor “evaluation time” and the interaction between factors “treatment and evaluation time” did not influenced the results. Figure 2B shows the relative metabolic differences of controls and experimental groups at each specific time point investigated (*Met*_*diff*_ 0, *Met*_*diff*_ 2, *Met*_*diff*_ 4 and *Met*_*diff*_ 6). The median values of relative metabolic activity in groups CHX, P90, P120 and P150 were observed to be negative, which indicates that these treatments were capable of reducing the biofilms’ metabolic activities. The median RLU values from groups CON, ARG and P30 were observed to be positive indicating that biofilms in these groups had their metabolic status upregulated by the conditions and treatments investigated.

**Table 3:** Median (minimum and maximum) values of antibacterial efficacy of plasma treatment on *S. mutans* biofilm in terms of metabolic activity.

Groups	Time				*p*-value
	Baseline	2 min	4 min	6 min	
Control	1.58 (− 0.15 a 2.92)	1.23 (− 0.18 a 3.61)	1.15 (− 0.09 a 4.38)	1.41 (− 0.46 a 4.28)	C
Plasma 30”	1.13 (0.07 a 6.47)	0.94 (− 0.03 a 4.69)	1.44 (− 0.05 a 3.51)	1.37 (− 0.24 a 5.24)	C
Plasma 90”	− 0.39 (− 0.72 a 0.11)	− 0.41 (− 0.78 a 0.51)	0.19 (− 0.78 a 0.39)	− 0.02 (− 0.84 a 0.67)	A
Plasma 120”	− 0.38 (− 0.57 a 0.08)	− 0.36 (− 0.65 a 0.11)	− 0.35 (− 0.58a − 0.04)	0.35 (− 0.65 a 0.26)	A
Plasma 150”	− 0.31 (− 0.80 a 0.43)	− 0.32 (− 0.68 a 0.31)	− 0.23 (− 0.68 a 0.11)	− 0.21 (− 0.68 a 0.55)	B
Argon gas	0.87 (− 0.07 a 6.04)	0.70 (− 0.31 a 5.85)	0.83 (− 0.22 a 5.32)	0.49 (− 0.22 a 3.35)	^a^
Chlorhexidine	− 0.83 (− 0.95 a − 0.54)	− 0.72 (− 0.95 a − 0.56)	− 0.66 (− 0.96 a − 0.46)	− 0.69 (− 0.94 a − 0.20)	*

Groups having similar letters are not significantly differences (*p* > 0.05), *Indicates significantly differences with all the groups, ^a^Indicates differences with P90, P120 and P150 groups.

**Figure 3:**
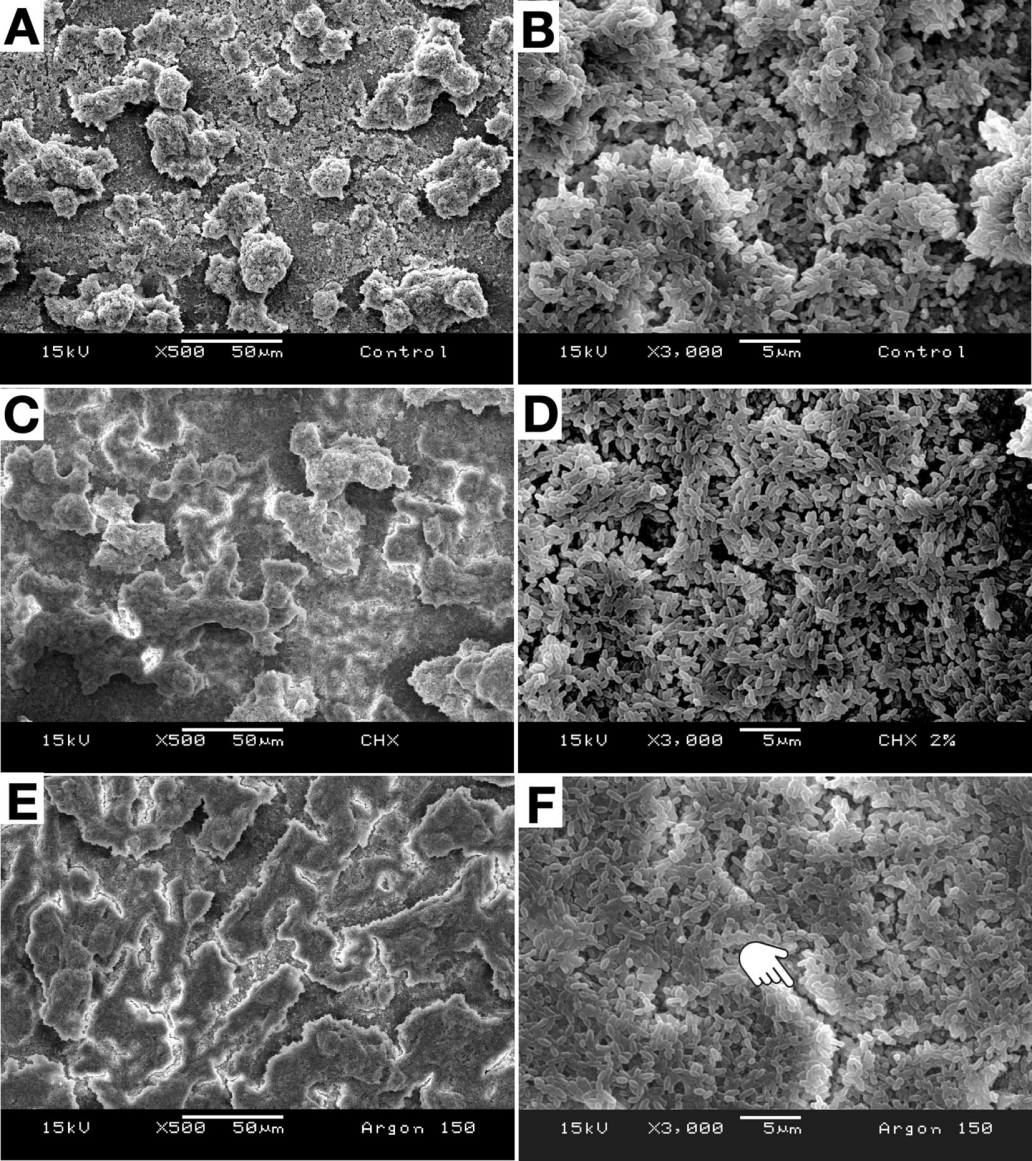
Representative SEM micrographs of the *S. mutans* biofilm (24 h, X500 and X3000 magnification) are shown after the following treatments: CON (**A**, **B**); CHX (**C**, **D**); and ARG (**E**, **F**). Note that the biofilm in ARG more compact appearance (**E**, **F**) when compare with the CON (**A**, **B**), some ruptures (**F**) occurred on the biofilm in ARG due to effects of drying (pointer).

**Figure 4:**
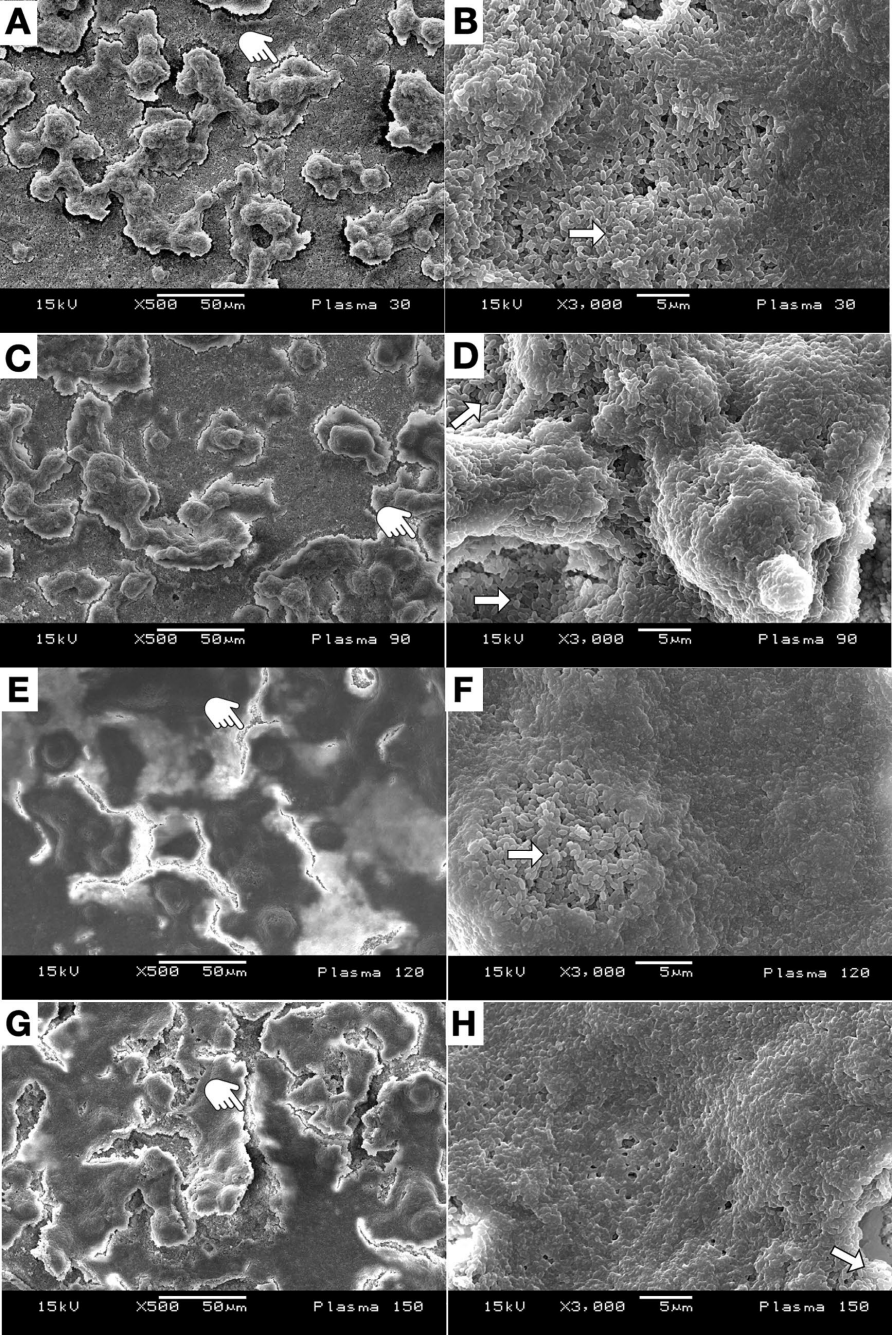
Representative SEM micrographs of the *S. mutans* biofilm (24 h, X500 and X3000 magnification), after NTAP treatment for P30 (**A**, **B**); P90 (**C**, **D**); P120 (**E**, **F**); and P150 (**G**, **H**). More damage on the biofilm structure was observed with the increase of the NTAP application. Note the presence of “non-disrupted” /untreated biofilm (white arrows) and some ruptures occurred on the biofilm due to effects of drying (pointer).

Representative SEM images of the antibacterial treatment effects on the morphological and structural features of *S. mutans* biofilms are shown in Figs. 3 and 4. The CON group displayed the typical structure and morphology of non-disrupted biofilms of *S. mutans* (Fig. 3A and B). The biofilm of CHX group was not experienced any adverse impacts on their structure or morphology as a consequence of treatment (Fig. 3C and D). For ARG treatment, biofilms seemed dehydrated as a direct consequence of argon gas jet (Fig. 3E and F).

When the biofilm was submitted to NTAP treatments, visible signs of degradation of the structure and morphology of *S. mutans* biofilms were noted. Also, it included localized damage, strong dehydration, and even complete disruption of cells. Regarding their architectures, biofilms observed were flattened as a consequence of NTAP treatments (Fig. 4).

**Figure 5:**
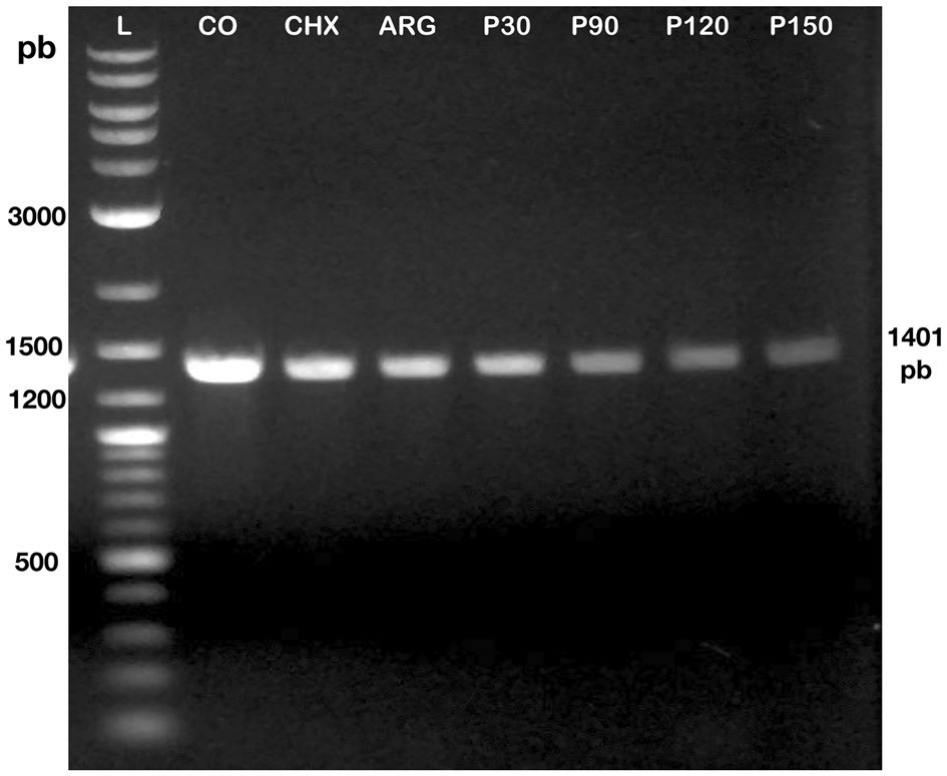
Agarose gel electrophoresis showing genomic DNA of *S. mutans* and PCR-amplified products of experimental groups.

For the PCR assay, the Fig. 5 shows that experimental groups treated with NTAP displayed bands (at 1401 pb) that were less bright than those of the groups CON, CHX and ARG, indicating the occurrence of NTAP-induced DNA damage.

## Discussion

This study showed that NTAP was effective against *S. mutans* biofilm grown on composite resin surface. NTAP reduced the viability of cells and their metabolic status, produced significant damage on the structure of investigated biofilms, and promoted genetic damage. Thus, the first null hypothesis that plasma would not have any antibacterial effect against biofilms of *S. mutans* was rejected.

The rationale for the selection of resin composite discs as a substrate to grow biofilms was based on the fact that more than five hundred million resin composite restorations are placed every year worldwide^51^ that these materials upregulate the aggregation and growth of oral bacterial^52^ and by-products generated by hydrolysis and biodegradation, typically change the ecology of biofilms from a state of health into a disease-associated state^53^.

Chlorhexidine and argon were used as control groups. The rationale for the selection of 2% chlorhexidine as a positive control for antibacterial effect is based on the fact the chlorhexidine is a broad-spectrum agent with proven activity against bacteria, some yeast and viruses^39,48,54^. On the other hand, argon gas was used as a control to determine if it had antibacterial effects on non-disrupted *S. mutans* biofilms. The reduction on the VCC showed by argon gas (Table 2) can be related to the drying effects on biofilm’s structure promoted by the argon air jet, and are according with other studies previously reported^36,55,56^. During the execution of antibacterial treatments, the humidity of specimens with biofilms was controlled by adding 20 µl of saline solution (each sample/every 60 s) to avoid any impact on cells’ viability due to dehydration phenomena. SEM images show ruptures and modifications on biofilm’s structure (Fig. 3E and F), that depending on the degree of dehydration, might adversely impact the structure and physical integrity of EPS^57^ precipitated by biofilms.

Previous studies have attributed NTAP’s antibacterial properties to a synergistic effect among RNS, ROS and UV-radiation^22,58,59^. It is believed that multiple interactions between free radicals (either RNS or ROS) and critical cellular components (membrane lipids, proteins, and DNA) results in lipid peroxidation and enzymatic activity disruption^21,35,58,60^. The combination of these factors leads to the oxidation of several amino acids from proteins and changes to the 3-dimensional structure of proteins. The cellular manifestation of these factors results in cells with compromised intracellular functions, that are not capable of maintaining their cytoplasmic pH levels, display unstable membranes and will eventually undergo inactivation due to the extravasation of intracellular contents^18,22^. Other studies have indicated that highly reactive gas radicals react with the surfaces of cells and living organisms through an “etching” mechanism^61,62^. In this physical process, follow-up by-products desorb from surfaces and create perforations in the membranes of microorganisms, thereby allowing secondary reactive species to adversely impact intracellular components, enzymatic reactions and DNA^63^.

The present study has comprehensively demonstrated that NTAP treatments investigated were effective against 24-h *S. mutans* biofilms. Treatments associated with longer exposure times resulted in higher bacterial reductions. The results reported in the present study are corroborated by previous investigations, Alkawareek et al. evaluated the potential cellular targets of NTAP and demonstrated that planktonic cultures of *Pseudomonas aeruginosa* displayed reduced viability (1 log_10_) after 30 s and were completely eradicated after 2 min of treatment with NTAP^62^. Li et al. investigated the effect of plasma treatment times (either 3 or 12 min) against biofilms of *Enterococcus faecalis* in root canals. Their results indicated that longer exposure times were associated with complete inactivation of biofilms^2^. Despite these reports, the results of the present study have shown that an antibacterial efficacy threshold is achieved after 100 s and further reductions in viability and metabolic status could not be observed (Fig. 1). These findings reject the second null hypothesis that plasma does not display time-dependent antibacterial properties.

The VCC results can be explained by NTAP’s mechanism of action where, during the first few seconds of NTAP treatment, critically damaged components of biofilms (cells, EPS, proteins, etc.) are deposited onto biofilms’ outermost layers, thereby reducing NTAP’s ability to penetrate into deep regions of biofilms. Previous reports have indicated that NTAP’s penetration depends on plasma composition, etching effectiveness and biofilm characteristics (e.g., thickness, composition, etc.)^20,59,62^. According to those studies, etching is more efficient against Gram-negative bacteria, and thicker biofilms typically present as a greater challenge for the penetration of plasma radicals. The bacterial inactivation results (viability reduction = 0.99 log, Table 2) observed in ARG were lower and significantly different (*p* < 0.05) when compared to the groups treated with NTAP, thereby allowing to infer that non-ionized argon gas does not display any antibacterial properties against non-disrupted *S. mutans* biofilm. Similar results using argon plasma jet on biofilms were also reported by other studies and could be explained by the drying effect of the gas flow^36,55,56^.

The metabolic activity of non-disrupted *S. mutans* biofilms was measured before and after NTAP treatments using a bioluminescence assay recently reported^45^. This assay is capable of determining, in a real-time and high throughput manner, the metabolic status of non-disrupted biofilms by the emission of visible light (530 nm) constitutively produced by cells. According to previous studies the amount of light produced is strongly correlated to bacteria’s intracellular energy potential and the total amount of viable cells^44,47,64^. Significant reduction of metabolic activity was obtained in groups with longer treatment times, which indicates that NTAP was able to immediately impact the metabolic status of non-disrupted *S. mutans* biofilms. Specimens in P30 displayed mean values of *Met*_*diff*_ (in RLUs) that were similar to those of CON and ARG groups, thereby indicating that NTAP treatments with short exposure times were not able to drastically downregulate the metabolic status of non-disrupted biofilms. It has been previously demonstrated that short NTAP treatment times results in concentrations of reactive species (either RNS or ROS) that are below the antibacterial threshold^56,62,65^. This suboptimal process results in weak oxidative stresses that increase bacterial respiration and intracellular ATP levels, and therefore, can be used to explain the unexpected high levels of metabolic activity observed in P30.

To investigate the hypothesis that NTAP treatments cause morphological and structural damage to biofilms and membranes of cells, an SEM analysis was performed. Figure 4 shows that NTAP treatments were capable of modifying the 3-dimensional structure of *S. mutans* biofilms in a time-dependent manner, where the increase in exposure time resulted in more severe structural damage. These findings are in agreement with previous publications and confirm the etching effects produced by NTAP treatments^61–63^, thereby rejecting the third null hypothesis that plasma does not display significant effects on the integrity of biofilms. Despite these promising results, Fig. 4F shows the presence of small regions displaying untreated biofilms. Such findings indicate that the manual sweeping application technique used during the delivery of NTAP is fundamentally important to ensure complete inactivation of biofilm.

Even though some studies showed that NTAP has limited effect on DNA^35,43,58,66^, the results presented on Fig. 5 indicate that NTAP treatments were capable of damaging DNA in a time-dependent manner, where longer exposure times yielded more DNA damage. These results are in agreement with the findings from the other experiments, thereby supporting the hypothesis that NTAP has a time-dependent antibacterial effect, and therefore, the fourth null hypothesis that plasma does not produce DNA damage on *S. mutans* biofilm was also rejected. Few studies have indicated that reactive oxygen species (including RNS and ROS) and photons emitted (UV radiation) by the NTAP could induce modifications on the nucleobases and thymine dimer formation, loss of DNA integrity^59,67^ and mutations^35,68^, thereby underscoring the need for further investigations on the effect of NTAP on persisted cells. However, in this context PCR have low power and other tests should be performed to confirm the results obtained in the present study.

The results of the present study have demonstrated that NTAP could be used as an alternative method for decontamination of restorative composite and other oral surfaces (both biotic and abiotic) prior to any treatment performed in the oral cavity. Among the numerous applications anticipated in dentistry, plasma could be used to disinfect cavity preparations, root canals^2,69,70^ and implant surfaces^71^ avoiding the problems associated with traditional methods including antibiotic resistance^15,16^, adversely impacts to adhesion procedures and longevity of polymer-based boned restorations^72,73^. Additionally, based on NTAP’s unspecific and multi-target mechanism of action, it is very unlikely that resistant strains of bacteria will be developed from the recurrent NTAP use^62,65^.

Even though the single-species biofilms evaluated in the present research are not relevant from the clinical stand point, the cariogenic model used in the present study has been utilized for many years to screen the efficacy of numerous antibacterial approaches^74^. This study represents a proof-of-concept effort to determine NTAP’s antibacterial efficacy against non-disrupted biofilms of *S. mutans* grown against the surfaces of relevant polymer-based restorative dental biomaterials. Futures studies using in situ multi-species biofilms should be performed to confirm the results of the present study.

## Conclusions

NTAP application for 150 s produced a reduction in bacterial viability of 38.3%. Bioluminescence results have also indicated that NTAP treatments are capable of immediately reducing the metabolic activity of non-disrupted *S. mutans* biofilm. SEM observations revealed that NTAP treatments induced significant changes onto the structure of biofilm. PCR results have shown that NTAP produced DNA damage. Thus, the findings of the present study indicate that NTAP can reduce the *S. mutans* biofilms on the surfaces of composite resins.
